# An inquiry into patient versus health system factors contribution to the diagnostic interval in oral cancer: an early diagnosis study from Kerala, India

**DOI:** 10.3332/ecancer.2024.1745

**Published:** 2024-08-22

**Authors:** Phinse Mappalakayil Philip, Srinivasan Kannan

**Affiliations:** 1 Department of Preventive and Community Oncology, Malabar Cancer Centre - Post Graduate Institute of Oncology Sciences and Research, Thalassery, Kerala, India; 2 Achutha Menon Centre for Health Science Studies, Sree Chitra Tirunal Institute for Medical Sciences and Technology, Trivandrum (An Institution of National Importance, Department of Science and Technology, Government of India)

**Keywords:** diagnostic interval, oral cancer, early diagnosis, diagnostic delay, health care system

## Abstract

**Introduction:**

Lip and oral cavity cancer is the second most frequent cancer in India, accounting for more than 10% of the total cancer incidence in the country. Oral malignancies are frequently found and diagnosed at advanced stages, resulting in dismal survival rates. The influence of healthcare-related factors in the diagnostic interval of oral cancer remains poorly understood.

**Methods and material:**

This study followed the principles of the Aarhus statement for early cancer diagnosis research. Researchers non-selectively recruited 261 patients with histopathologically proven Squamous Cell Carcinoma of the oral cavity at the comprehensive Cancer Care Centre in Northern Kerala, India. They acquired information in direct patient interviews using validated instruments. They triangulated self-reported data with case notes, referral letters and biopsy results.

**Results:**

The median (Interquartile range) diagnostic interval reported by the study participants (*n* = 261) was 36.00 (14.00–76.50) days. The proportion of participants having diagnostic intervals of more than 30 days was 57.9% (*n* = 151). The predictors of diagnostic interval include ‘Type of advice provided by the health care provider’, Number of healthcare providers consulted in the diagnostic journey, ‘Age of the participant’, ‘Monthly income’ and ‘Caste’.

**Conclusion:**

Nearly three-fifths of the study participants had diagnostic intervals that exceeded the acceptable limit, highlighting the need to streamline the facilities and processes required for early diagnosis of oral cancer. Strengthening the health system at the primary level by incorporating referral guidelines and in-service training of primary care practitioners will reduce diagnostic intervals for oral cancer.

## Introduction

Oral cancer is India’s second most frequent cancer, accounting for 10.3% of total cancer incidence in 2020 [1]. During the same era, India accounted for more than one-third of global oral cancer incidence. More than two-thirds of all oral malignancies in the world were diagnosed at advanced stages [2]. Detecting oral cancer in its late stages often results in a poor prognosis [3]. The most critical step in improving survival rates is to diagnose the condition early on [4]. The diagnostic interval, also known as diagnostic delay, provider delay or professional delay, is the time between the initial consultation with a healthcare provider and the definitive histological diagnosis [5]. Evidence suggests an inverse relationship between diagnostic interval duration and cancer-related mortality [6]. A thorough understanding of the length of the diagnostic interval and its contributing variables is critical for shortening the diagnostic journey in oral cancer. This will aid in the effective utilisation of current cancer diagnostic and management facilities in the health system. Previous research has demonstrated that a variety of patient and healthcare system characteristics influence the length of the cancer diagnostic journey [5]. The available literature on early cancer diagnosis is primarily from Western countries, where the health infrastructure and cancer incidence patterns differ from those of developing countries such as India. There is a lack of understanding of several aspects of early oral cancer detection in South Asian countries, especially India, where the bulk of mouth cancer incidence and death have been documented in the world [7]. Fewer studies have been undertaken to determine the duration of the diagnostic gap and the many factors that contribute to it in oral cancer. Furthermore, the majority of the early cancer diagnosis studies described in the available literature were not conducted in compliance with the principles specified in the Aarhus Statement, making comparisons between the studies problematic [5]. The present study assessed the role of various healthcare system-related factors and other sociodemographic factors in the diagnostic journey of oral cancer. The current study sought to assess the length of time to diagnosis of oral cavity cancers, as well as the many factors that contribute to it.

## Methods and materials

The current study on diagnostic intervals in oral cancer was part of a larger study at a Comprehensive Cancer Centre in northern Kerala, India, from December 2019 to August 2020. The study was approved by the Institutional Ethics Committee (1617/IRB-IEC/13/MCC/13-05-2019/5) of the institution. The design as well as the reporting of the study followed the Aarhus statement for early cancer diagnosis research [5]. The detailed study protocol has been published elsewhere [7]. The project was divided into three phases: development and validation of data collection tools, a hospital-based cross-sectional survey and stakeholder interviews. The details of the data collection tool development process were given elsewhere [8]. A brief overview of the tool development process includes: 1) a Literature review and the development of an inventory, 2) Consultation with experts in the field of cancer control, 3) Content validity assessment using the Content Validity Index, 4) Translation and back translation into the local language, 5) Assessment of face validity, 6) Evaluation of the questionnaire by the Technical Advisory Committee and 7) Evaluation of the questionnaire by the Institutional Ethics Committee. The newly developed tool consisted of questions to identify two-time points in the diagnostic journey of oral cancer, namely, the ‘Date of the first presentation at a healthcare facility’ and the ‘Date of diagnosis’. These time points are necessary to calculate the diagnostic interval and thereby the diagnostic delay. The ‘Date of first presentation’ is defined as ‘the time point at which, given the presenting signs, symptoms, history and other risk factors, it would be at least possible for the clinician seeing the patient to have started an investigation or referral for possible important pathology, including cancer’ [5]. The ‘Date of diagnosis’ was determined using the ‘Hierarchy for Defining the Date of Diagnosis’ provided by the European Network of Cancer Registries. Priority was given to the date of the biopsy [5]. Many patients fail to recall the exact dates of events in their mouth cancer diagnosis journey. In such a case, the only option is to calculate a pseudo-exact date based on the patient’s stated estimate. Neal *et al* [9] presented protocols for calculating and validating pseudo-exact dates based on the estimated dates given by the patient. These two protocols were used in our study after being adapted to the local context, considering the seasonal and cultural aspects of our country [8, 9].

We used the following inclusion-exclusion criteria for the cross-sectional study. Newly registered patients with malignant neoplasms of the lip and oral cavity (ICD Code C00 –C06) were included in the study. The exclusion criteria include. 1) Those known to have or had other cancers, 2) Patients who were on routine surveillance for cancer, 3) Those who were not consenting to participate, 4) Oral cancer patients who were unable to participate due to health reasons or any other reasons, 5) Oral cancer patients with recurrence and 6) Patients who have completed treatment for oral cancer. Oral cancer patients who reported to the institution during the study period and met the inclusion-exclusion criteria were recruited in sequence until the required sample size was reached [7]. The first author conducted direct patient interviews using the newly developed and validated questionnaire. Participants who consented to participate in the study were interviewed by the investigator during one of the follow-up visits. The interviews were performed at a place and time convenient for the patient, within the hospital. The interviews lasted for approximately 30 minutes. To reduce information bias, patient interviews were conducted within 3 months after diagnosis.

Data Analysis: Categorical variables were described in terms of frequencies and proportions. Mean and standard deviation were calculated for continuous variables normally distributed. Non-normally distributed variables were described in terms of median and interquartile range. Bivariate analysis of categorical variables was performed with the help of a contingency table and chi-square statistics or Fischer’s exact test. Binary logistic regression analysis was carried out to formulate a predictive model of the association between diagnostic interval and possible predictor variables. One month is considered an acceptable duration for the diagnostic interval [10] for analysis purposes. The outcome variable, the diagnostic interval was dichotomized into ‘Less than or equal to 30 days’ and ‘More than 30 days’ and binary logistic regression analysis was performed.

## Results

The study included 261 oral cancer patients who reported to the Comprehensive Cancer Care Centre during the study period from December 2019 to August 2020. Nearly one-third (29.1%, *n* = 76) of the participants were females. One-tenth (10.7%) of the participants belong to the scheduled tribe. The mean age of the respondents was 60.77 ± 12.3 years, ranging from 33 to 95 years. The majority of the participants were Hindus (71%), followed by Muslims (21%) and Christians (8%). If we look at the caste affiliation of the participants, the majority were from the Other Backward Class of the community (64%) and 18% belonged to the General category. One-tenth (10.7%) of the participants belong to the scheduled tribe. Nearly one-fifth (19.9%) of study participants were either widows or separated or divorced, and nearly three-fourths (74.3%) of the participants lived in nuclear families. More than three-fourths lived in the Panchayath area (81.6%), and the rest were from urban areas like municipalities and corporations. Panchayath, municipality and corporation are local administrative units in India. Among study participants, 28.4% did not have formal school education. The majority (64.4%) of the participants were daily wagers, and more than two-thirds of the participants (73.9%) belonged to the BPL (Below Poverty Line) category. More than half of the participants (58.2%) reported a monthly income of 3,000 to 10,000 Indian rupees. The median (IQR) income of respondents was 5,000 (3,400–9,000) INR per month.

The median (interquartile range) diagnostic interval reported by study participants (*n* = 261) was 36.00 days (14.00–76.50). The median (IQR) diagnostic interval for males was 36.00 (13.75–81.50) days, and for females, it was 36.00 (14.00–65.00) days. The proportion of subjects with a diagnostic interval greater than 30 days was 57.9% (*n* = 151). In the bivariate analysis, sociodemographic characteristics such as caste (*p* = 0.021) and marital status (*p* = 0.042) (Table 1) and healthcare-related factors like ‘Advice from a Health Care Provider (HCP) at the first consultation’, ‘The number of HCPs consulted (route to diagnosis) before receiving a definitive cancer diagnosis’ and ‘The type of HCP with whom the patient first consulted to discuss the current problem in the oral cavity’ were found to be significantly associated with the diagnostic interval in oral cancer (Table 2)

The binary logistic regression analysis has identified five detrimental factors for diagnostic delay. Of these, three were patient-related and two were health system-related. The predictors include ‘Monthly income’, ‘Type of advice provided by the healthcare provider’, ‘The number of healthcare providers consulted in the diagnostic journey’, ‘Age of the participant’ and ‘Caste’ (Table 3). The patient-related factors identified in the model were largely non-modifiable. They include being under the age of sixty, belonging to a scheduled tribe or general caste, and having a monthly income of less than 5,000 Indian rupees (approximately 60 US dollars). Unlike the patient-related factors, the health system-related factors identified as detrimental in the study are modifiable. Consulting with three or more healthcare providers for a symptom suggestive of cancer will increase the risk of a prolonged diagnostic interval. The establishment of proper cancer-specific referral guidance in the healthcare system will avoid multiple healthcare provider consultations in the diagnostic journey. The advice or guidance provided by the healthcare provider with whom the patient consulted for the symptom suggestive of cancer is an important factor that determines the course of the diagnostic journey. When the health provider dismisses the presenting symptom as minor or insignificant, the diagnostic interval increases. On the other hand, a prompt referral to higher centers or advice for a biopsy will reduce the diagnostic interval. A low index of suspicion for symptoms suggestive of oral cancer invariably increases the length of the diagnostic interval. Cancer site, cancer stage and the presence of pre-existing co-morbidities were not significantly associated with the diagnostic interval in our study. The association of various tobacco and alcohol habit-related factors with the diagnostic interval were analysed, but no significant association was observed. Access to the nearest healthcare facility, such as transport options, distance to the healthcare facility, time taken to reach the nearest health center and so on, were not found to be significantly associated with the diagnostic interval. Similarly, the medical or dental consultation pattern and the first response to general health problems were also not associated with diagnostic intervals.

## Discussion

The median diagnostic interval reported in our study was 36 days and the proportion of participants having increased diagnostic interval more than 30 days was nearly three-fifth. Existing literature suggests diagnostic interval of more than 1 month can lead to an advanced stage of cancer presentation and poor survival rates [11, 12]. The duration of the median diagnostic interval and the proportion of participants having increased diagnostic interval varies considerably across studies. The median diagnostic interval of 21 days [13], 30 days [14] and 86 days [15] were reported in studies from UK, India and Iran, respectively.

Although the socioeconomic position is an independent predictor of survival and stage of diagnosis in oral cancer [16], only a few sociodemographic and economic characteristics including caste, marital status and monthly income were identified to influence the diagnostic interval in our investigation. Compared to married adults, those who were ‘single’ were more likely to report a diagnostic interval longer than 30 days. This could be due to the married person’s support from their spouse and family. Previous studies on the delay in diagnosis of breast cancer have found a strong relationship between the diagnostic interval and marital status [17, 18]. A meta-analysis of the association of social networks and cancer mortality found that unmarried people are at a higher risk of death [19]. The protective effect of marriage on cancer survival has already been reported in the literature [20]. Another predictor for prolonged diagnostic interval observed in this study was ‘Caste’. In India, ‘Caste’ refers to social standing and socioeconomic position [21]. Caste also affects individuals’ access to education, employment and healthcare [22]. Lower caste people are subjected to social and economic exclusion [23]. A previous study on the treatment-seeking behavior of the tribal population in India reported poor healthcare utilisation among tribal people [24]. The larger diagnostic interval reported in general caste persons may be related to the low suspicion of oral cancer among those belonging to better socioeconomic strata, as mouth cancer is a disease of the poor [25, 26]. However, further studies are necessary to validate this observation. In this study, participant age was found to be a significant predictor of the diagnostic interval. Participants under 60 had longer diagnostic intervals compared to those over 60. Similar outcomes were reported in studies from Iran and India [14, 27]. This may be linked to a lower index of oral cancer suspicion by healthcare professionals among patients belonging to younger age groups, as oral cancer is more common in older persons [28]. In binary logistic regression analysis, monthly income was also a significant predictor of diagnostic interval. A systematic review of factors affecting therapeutic compliance identified income and cost of treatment as determining factors [29]. A study from Iran similarly found that family income was connected with diagnostic delays in breast cancer [17]. In this study, those participants who first consulted a general medical or dental practitioner had shorter diagnostic intervals than those who first consulted an alternative medicine practitioner. A study of breast cancer patients in Bangladesh observed a relationship between alternative medicine use and delayed treatment seeking [30]. Ayurveda, Yoga and Naturopathy, Unani, Siddha, Sowa-Rigpa and Homeopathy are the alternative systems of medicine recognised by the Government of India [31]. According to a nationally representative survey, registered alternative medicine practitioners provided 6.9% of all outpatient services in India, with particularly high utilisation in Kerala [31]. This highlights the necessity to teach alternative medicine practitioners in the early detection of oral cancer. The nature of initial advice or instruction given by the HCP first consulted was identified as a predictor of the diagnostic interval. A study from the United Kingdom on general practitioners’ initial response to symptoms suggestive of oral cancer highlighted the need for streamlining the oral cancer diagnostic pathway to minimize diagnostic delay, as one-quarter of the study participants did not receive appropriate instructions for subsequent management [32]. HCPs in the first point of contact in the health system should be sufficiently trained to identify the early signs and symptoms of oral cancer. In a study from the UK, only 15% of the medical practitioners and 37% of dental surgeons considered themselves confident in identifying oral premalignant and malignant lesions [33]. In our study, consulting with three or more HCPs in the diagnostic journey was found to be a significant predictor for having prolonged diagnostic intervals. A systematic review observed that oral cancer patients on average had to make three consultations before referral to a specialist [34]. Macpherson *et al* [33] studied the referral pattern of primary health care professionals in oral cancer diagnosis and observed that more than half of the medical and dental HCPs re-evaluated their patients before referring them to higher centers. In a previous study, 23% of medical practitioners and 48% of dental surgeons expressed confidence in their ability to decide on urgent referrals for oral lesions [33]. Another reason for multiple routes to diagnosis was the absence of an established referral system. We recommend the following future steps to improve the diagnostic interval in oral cancer (Table 4).

## Conclusion

Nearly three-fifths of the study participants had diagnostic intervals that exceeded the acceptable threshold, emphasizing the importance of streamlining the facilities and processes needed for the early detection of oral cancer. Several health-care system elements as well as patient-level characteristics influence the diagnostic journey for oral cancer. The low index of suspicion for oral cancer in primary care extends the diagnosis interval. Strengthening the primary healthcare system by implementing referral guidelines and providing in-service training to primary care practitioners will shorten the diagnostic interval for oral cancer. The remaining patient factors, such as age, caste and income, are largely unmodifiable, and structural factors must be addressed to reduce their impact on the diagnosis interval.

## Conflicts of interest

All authors report no relationships that could be construed as a conflict of interest.

## Funding

The authors state that no external funding was received for this study. In addition, the authors certify that this work has no financial conflicts of interest.

## Author contributions

The authors confirm their contribution to the paper as follows. Study conception and design: PMP, SK, data collection PMP; analysis and interpretation of results: PMP, SK. draft manuscript preparation PMP, SK. All authors reviewed the results and approved the final version of the manuscript.

## Figures and Tables

**Table 1.** Sociodemographic factors associated with diagnostic interval in oral cancer patients (*n* = 261).

Variable	Diagnostic interval ≤ 30 days *n* (%)	Total > 30 days *n* (%)	Chi-square test *n* (%)	*p*-value
**Age**				
≤60 years	50 (36.8)	86 (63.2)	136 (100)	0.066
> 60 years	60 (48.0)	65 (52.0)	125 (100)	
**Sex**				
Female	27 (35.5)	49 (64.5)	76 (100)	0.165
Male	83 (44.9)	102 (55.1)	185 (100)	
**Caste**				
General	15 (31.9)	32 (68.1)	47 (100)	0.021^*^
Other Backward Caste	81 (48.5)	86 (51.5)	167 (100)	
Scheduled Caste	8 (42.1)	11 (57.9)	19 (100)	
Scheduled Tribe	6 (21.4)	22 (78.6)	28 (100)	
**Income**				
≤5,000 rupees	53 (38.1)	86 (61.9)	139 (100)	0.161
> 5,000 rupees	57 (46.7)	65 (53.3)	122 (100)	
**Marital status**				
Married	90 (45.7)	107 (54.3)	197 (100)	0.042^*^
Single	20 (31.3)	44 (68.8)	64 (100)	
**Total**	**110 (42.1)**	**151 (57.9)**	**261 (100)**	

* *p-value* less than 0.05

**Table 2.** Association of healthcare-related factors and diagnostic interval in oral cancer patients (*n* = 261).

Variable	Diagnostic interval ≤ 30 days *n* (%)	Total > 30 days *n*(%)	Chi-square test *n* (%)	*p*-value
**Type of HCP first met**				
Medical doctor	29 (33.3)	58 (66.7)	87(100)	
Dental surgeon	33 (44.6)	41 (55.4)	74 (100)	< 0.001^*^
Other medical specialist	47(58.8)	33 (41.3)	80 (100)	
Other systems of medicine	1(5.0)	19 (95.0)	20 (100)	
**Advice by HCP**				
Ignored symptom	10 (12.3)	71 (87.7)	81 (100)	< 0.001^*^
Advised biopsy	61 (59.8)	41 (40.2)	102 (100)	
Referred to higher center	39 (50.0)	39 (50.0)	78 (100)	
**Number of HCPs consulted in the diagnostic pathway**				
One	27 (75.0)	9 (25.0)	36 (100)	
Two	51 (54.8)	42 (45.2)	93 (100)	
Three	20 (25.6)	58 (74.4)	78 (100)	< 0.001^*^
Four & above	12 (22.2)	42 (77.8)	54 (100)	
**Total**	**110 (42.1)**	**151 (57.9)**	**261 (100)**	

HCP – Health Care Provider

* *p-value* less than 0.05

**Table 3.** Binary logistic regression analysis of predictors of diagnostic interval.

Variable	Frequency	Adjusted odds ratio (95% CI)	*p*-value
**Age**			
≤60 years	136	**2.090 (1.1-3.9)**	**0.020**
>60 years	125	Reference	
Caste			
Other backward class	167	Reference	
General class	47	**2.681 (1.2-6.2)**	**0.021**
Scheduled caste	19	1.412 (0.4-4.5)	0.558
Scheduled tribe	28	**3.85 (1.3-11.1)**	**0.012**
**Number of HCPs consulted in the diagnostic pathway**			
One	36	Reference	
Two	93	2.533 (0.98-6.5)	0.054
Three	78	**6.593 (2.3-19.0)**	**0.001**
Four & above	54	**4.743 (1.5-15.5)**	**0.010**
**Advice by HCP**			
Ignored symptom	81	**5.716 (2.3–14.3)**	**0.001**
Advised biopsy	102	0.700 (0.4-1.4)	0.296
Referred to higher center	78	Reference	
**Monthly income**			
≤ 5000 rupee	139	**2.681 (1.2–6.2)**	**0.021**
> 5,000 rupees	122	Reference	

Controls were ‘Comorbidities’ and ‘Marital status’ and they were excluded from the table

*p-value* less than 0.05

**Table 4.** Recommendations for future action to reduce diagnostic delay in oral cavity cancers.

S/N	Recommendations
1	Healthcare institutions should systematically collect the duration of diagnostic interval for each patient and steps should be taken to reduce the duration to less than 30 days for all patients.
2	The health system should be strengthened at the primary care level by providing in-service training to facilitate early diagnosis of oral cancer.
3	The health system should have a cancer-specific referral pathway.
4	Oral health care facilities should be established at primary care to facilitate opportunistic oral cancer screening.
5	All general medical and dental practitioners should be trained in tobacco cessation strategies.
6	Workplace and community-based oral cancer awareness activities should be held regularly to familiarize the population with the early warning signs and symptoms of mouth cancer.
7	Diagnostic delay is high among the tribal population. Special efforts should be made to improve access to oral health care among the tribal population.
8	Healthcare facilities in remote locations should have extended working hours so that individuals can use these services after their regular working hours.
9	Practitioners of AYUSH and other systems of medicine should also be given training in early identification of signs and symptoms of oral cancer.
10	Patient navigation programs may be implemented in rural areas to facilitate adherence to follow-up instructions.
